# Real-World Video Super-Resolution with a Degradation-Adaptive Model

**DOI:** 10.3390/s24072211

**Published:** 2024-03-29

**Authors:** Mingxuan Lu, Peng Zhang

**Affiliations:** School of Electronics and Communication Engineering, Shenzhen Campus of Sun Yat-Sen University, Shenzhen 518107, China; lumx7@mail2.sysu.edu.cn

**Keywords:** real-world video super-resolution, degradation-adaptive, pre-cleaning, lightweight network, deep learning

## Abstract

Video super-resolution (VSR) remains challenging for real-world applications due to complex and unknown degradations. Existing methods lack the flexibility to handle video sequences with different degradation levels, thus failing to reflect real-world scenarios. To address this problem, we propose a degradation-adaptive video super-resolution network (DAVSR) based on a bidirectional propagation network. Specifically, we adaptively employ three distinct degradation levels to process input video sequences, aiming to obtain training pairs that reflect a variety of real-world corrupted images. We also equip the network with a pre-cleaning module to reduce noise and artifacts in the low-quality video sequences prior to information propagation. Additionally, compared to previous flow-based methods, we employ an unsupervised optical flow estimator to acquire a more precise optical flow to guide inter-frame alignment. Meanwhile, while maintaining network performance, we streamline the propagation network branches and the structure of the reconstruction module of the baseline network. Experiments are conducted on datasets with diverse degradation types to validate the effectiveness of DAVSR. Our method exhibits an average improvement of 0.18 dB over a recent SOTA approach (DBVSR) in terms of the PSNR metric. Extensive experiments demonstrate the effectiveness of our network in handling real-world video sequences with different degradation levels.

## 1. Introduction

Sensors play a crucial role in numerous aspects of our everyday lives, finding applications in diverse fields such as environmental monitoring, traffic management, medical health, and robotics [1,2,3]. Although video sensors have been designed to operate efficiently in environments with a low latency and complexity, they continue to impose restrictions on the quality of input videos. In order to tackle this challenge, various video processing techniques have been employed for restoration, including video super-resolution [4,5,6,7,8] and video denoising [9,10,11]. These methods strive to improve the overall quality of video input, ensuring clearer and more refined outputs across different applications.

Video super-resolution (VSR) is an active area of research within the visual domain, which aims at enhancing the resolution of low-resolution (LR) videos [12]. Due to the introduction and application of convolution neural network (CNN) methodologies, the development of VSR algorithms has been progressing. For instance, in the pioneering work of SOFVSR [13], the motion-compensated low-resolution input undergoes processing within an optical flow reconstruction network, ultimately generating high-resolution video frames. TDAN [7] implicitly integrates motion information into low-quality frames, which can be extracted without computing the optical flow. BasicVSR uses a bidirectional propagation network, which relies on optical flow alignment and is employed to aggregate information across the spatio-temporal dimension [6]. However, most of these ignore complex degradation processes in the real world. They assume an ideal degradation process (bicubic downsampling) from HR to LR [14], which leads to performance drops when processing real-world videos because of mismatched degradation. In contrast, blind video super-resolution aims to restore low-resolution images suffering from unknown and complex degradations [15]. The remarkable work of Real-ESRGAN enhances the image perceptual quality, which introduces complex degradation operations including noise, blur, and down-sampling [16]. However, its recovery ability is limited by the practical usage. In particular, it generates better details in severely degraded LR inputs than in mildly degraded LR inputs.

Considering the aforementioned challenges, we propose a novel video super-resolution framework (DAVSR), which adaptively restores video sequences with varying degrees of degradation. Similar to Real-ESRGAN, our goal is to improve the quality of real-world low-resolution (LR) videos through synthesizing training pairs with a practical degradation process. Real-ESRGAN solely employs second-order degradation processes to synthesize its training data [16], lacking the ability to effectively capture the characteristics of mildly degraded low-resolution (LR) videos. This motivates us to expand the degradation model into an adaptive degradation model to restore LR videos degraded to various degrees. Specifically, we use “first-order” degradation modeling to synthesize training pairs for mildly degraded LR videos and “high-order” degradation modeling for severely degraded videos. The “first-order” degradation results from complex combinations of different degradation processes, including camera blur, sensor noise, and video compression. The “high-order” degradation is represented through the application of multiple repeated degradation operations.

Recently, Huang et al. [17] showed that temporal redundancy brings adverse effects to the information propagation in most existing VSR methods. For example, this information also leads to exaggerated artifacts, arising from the accumulation of errors during propagation. As a result, we used an image pre-cleaning module [18,19,20] to reduce the adverse effects of noise and artifacts on propagation. Furthermore, to establish accurate connections among multiple frames, we have integrated an unsupervised optical flow estimator to make the most of its potential. Finally, to balance performance and the computational cost, we simplified the propagation network and reconstruction module of BasicVSR, further reducing model complexity.

The primary contributions of this work are outlined below:

- We propose a degradation-adaptive process to model more practical degradations, which notably boosts the model’s capability to enhance the resolution of videos affected by various degradation levels. It can attain a superior visual performance compared to prior works, making it more applicable in real-world scenarios.
- Moreover, we present an exquisite image pre-cleaning module for removing degradations in the input images prior to information propagation. It can reduce the adverse effects of temporal redundancy in video super-resolution.
- Finally, the introduced unsupervised optical flow estimator mitigates the inaccuracies in optical flow encountered with previous methods. Furthermore, a new lightweight reconstruction block is proposed to further reduce the complexity. Many experimental results validate the superiority of the proposed method over state-of-the-art methods.

The remainder of this paper is structured as follows: Section 2 introduces some related works on video super-resolution. In Section 3, the proposed DAVSR network is described in detail. Section 4 demonstrates the experimental results of our method. Finally, the conclusions and future work are presented in Section 5.

## 2. Related Work

### 2.1. Real-World Video Super-Resolution

The goal of video super-resolution is to reconstruct high-resolution video frames from the low-resolution ones. Most existing VSR methods are trained with predefined degradations, such as bicubic downsampling. However, they exhibit significant deterioration when confronted with unknown degradations in practical applications. Meanwhile, scaling from VSR to real-world VSR is not trivial due to the difficulty of modeling complex degradations in the wild. To solve this problem, recent blind super-resolution [14,15,21,22] proposes a solution where the inputs are assumed to be degraded through a recognized process characterized by unknown parameters. Specifically, they train the network through a predefined process with unknown parameters. Despite the fact that this network can restore some degraded videos, it is still insufficient for real-world generalization. Fortunately, Wang et al. proposed a second-order degradation process to simulate complex degradation, including blur, noise, compression, and other factors. These methods exhibit an encouraging performance in real-world super-resolution. However, we have observed that they generate better details in severely degraded low-resolution (LR) inputs compared to those with mild degradation. In this work, we explore the process of degradation modeling and propose a novel video super-resolution framework, which adaptively recovers videos with different levels of degradation.

### 2.2. Optical Flow Estimation

To explore the temporal dependency between consecutive video frames, numerous video super-resolution methods based on deep learning estimate optical flows from low-resolution (LR) frames for motion compensation. The quality of the optical flow directly influences the efficacy of these methods. With the advancements in deep learning, some optical flow estimation methods trained by CNN networks achieve better results than non-learning methods. The model’s generalization ability is constrained by the domain difference between the optical flow of the synthetic dataset and the real-world dataset. Therefore, Yu et al. [23] initially proposed an unsupervised method for optical flow learning, emphasizing brightness constancy and motion smoothness. Wang et al. [24] propose using an unsupervised optical flow estimator to bypass the necessity for labeled data. Further, for the problem of large motion and model occlusion, they proposed a new warping module to improve the performance of the unsupervised optical flow method. Although these methods [23,24,25] have become sophisticated, the estimated optical flow accuracy is still worse than that of SOTA supervised methods. In addition, they have not solved the difficulty of estimating an accurate optical flow from severely degraded inputs. We enhance the quality of the low-quality (LQ) flows by employing a knowledge distillation approach to learn high-quality (HQ) image characteristics.

### 2.3. Image Pre-Cleaning

The effectiveness of existing video super-resolution algorithms largely relies on leveraging temporal information from adjacent frames. Inspired by Huang et al. [17], the introduction of temporal redundancy can negatively impact information propagation. Therefore, it is crucial to eliminate degradations before propagation which is beneficial for suppressing artifacts in the outputs. While analogous concepts have been discussed within the realm of single-image super-resolution (SISR) [18,19,26,27], they have not undergone a thorough exploration in the realm of VSR. Furthermore, we devise a dynamic pre-cleaning process for video frames with different levels of degradation. For mildly degraded low-quality video sequences, we can use a cleaning strategy once to remove redundant temporal information. Excessive cleaning can result in the loss of valuable information. In particular, the temporal redundancy introduced by the degradation process in video sequences is mitigated through the utilization of a pre-cleaning module prior to network propagation. A series of ablation experiments were conducted to confirm the effectiveness of our pre-cleaning module.

## 3. Proposed Method

**Figure 1:**
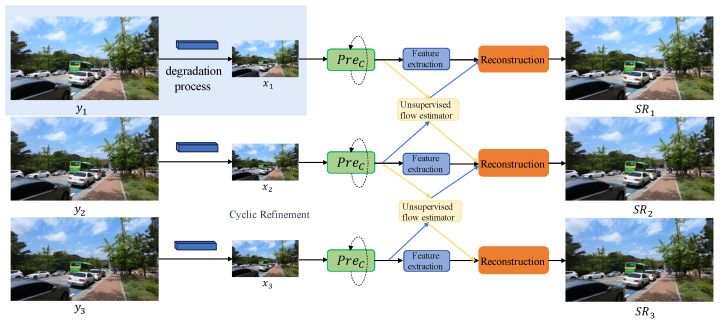
The architecture of the DAVSR network, when the number of input frames N = 3. Degradation process: aims to generate low-resolution frames.

In this section, we propose an unsupervised flow-aligned degradation-adaptive network for real-world video super-resolution, which we have denoted as DAVSR. As depicted in Figure 1, the proposed method is composed of four modules: the degradation-adaptive module, the image pre-cleaning module, the feature alignment module, and the reconstruction module. Let $y_{i}$ represent the input video sequence, which go through a degradation module to generate $x_{i}$, the low-quality video sequence derived from $y_{i}$. Subsequently, $x_{i}$ passes through an alignment and reconstruction module to yield the super-resolution video sequence $SR_{i}$.

In the following sections, the details of the proposed module are introduced.

### 3.1. The Degradation-Adaptive Model

When the classical degradation model is employed to synthesize training pairs [28], it becomes challenging to acquire high-quality real-world LR–HR image pairs. The network model exhibits limitations in learning from image pairs that faithfully represent real-world scenarios, leading to suboptimal performance in the restoration of corrupted video sequences under real-world conditions. The classical degradation model incorporates typical fundamental degradations, such as blur, resize, and compression, and can be considered as first-order modeling. However, degradation processes in real-world scenarios are highly diverse, comprising a multitude of steps such as camera imaging systems, internet transmission, etc. Inspired by Wang et al. [16], it becomes evident that a complex degradation process cannot be effectively modeled with a first-order approach. A higher-order modeling process is proposed to simulate the intricate degradation processes found in the real world, denoted by *S*. The degradation space *S* is composed of a series of degradation process $D=[D_{1},D_{2},...,D_{n}]$. Specifically, our method incorporates a range of degradation operations, including noise (both Poisson noise and Gaussian noise) [29], various forms of blurring (isotropic, anisotropic, Gaussian filter), resizing (both bicubic and bilinear), and JPEG and video compression. As in Equation (1), let *y* stand for the original high-resolution (HR) frame, where *D* represents a set of various degradation operations and *X* corresponds to the derived low-resolution (LR) frame after undergoing degradation. Depending on the specific application scenarios, customization of the degradation process is feasible, such as the inclusion of additional noise or adjustments in degradation factor parameters.

(1) $$X=D^{n}\left(y\right)=\left(D_{n}...D_{2}D_{1}\right)\left(y\right)$$

**Figure 2:**
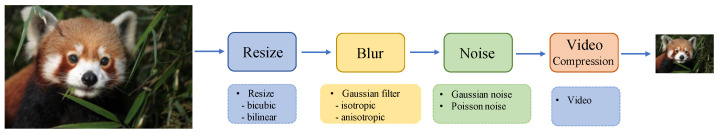
The $S_{1}$ degradation process. The $S_{3}$ degradation process involves two repeated degradation processes.

In contrast to Real-ESRGAN, we have introduced video compression into the degradation process. This is because video compression implicitly takes into account the spatio-temporal information of video frames, bringing benefits to the degradation. While the second-order degradation process utilized in Real-ESRGAN can generate an extensive degradation space, effectively adapting to videos with diverse degradation levels remains challenging. Hence, we divide the degradation space *S* into three levels, represented as $\left[S_{1},S_{2},S_{3}\right]$, by configuring the parameters *D* accordingly. Among these, $S_{3}$ is generated by second-order degradation, while $S_{1}$ and $S_{2}$ are, respectively, produced by first-order degradation processes with different parameters. As depicted in Figure 2, the $S_{1}$ degradation process is visible. The $S_{3}$ degradation space corresponds to videos with severe degradation, the $S_{2}$ degradation space corresponds to videos with moderate degradation, and the $S_{1}$ degradation space corresponds to mild degradation. Therefore, our degradation space *S* better captures real-world degradation processes.

### 3.2. The Image Pre-Cleaning Module

**Figure 3:**
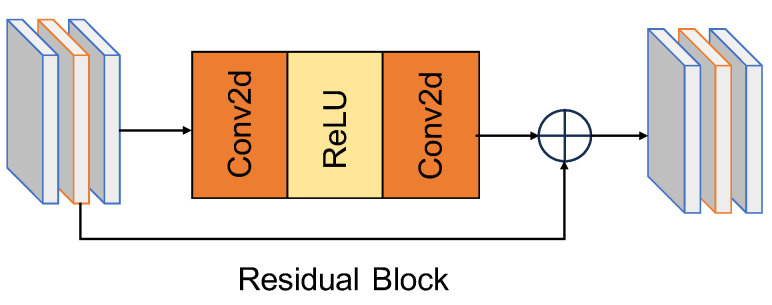
The pre-cleaning module consists of 15 cascaded residual blocks.

Inspired by Huang et al. [17], the introduction of temporal redundancy can have adverse effects on information propagation. Therefore, removing degradations prior to propagation is crucial, as it aids in suppressing artifacts in the outputs. A simple pre-cleaning module is proposed to suppress degradation; its effectiveness is shown by the experimental results. Our pre-cleaning module consists of 15 cascaded residual blocks. The residual blocks are illustrated in Figure 3.

The degraded images are passed to the pre-cleaning module to remove redundant temporal information. $x_{i}$ represents the *i*-th image of the input sequence, ${\hat{x}}_{i}$ is the clean image, and $Pre_{C}$ is pre-cleaning module, as in Equation (2).

(2) $${\hat{x}}_{i}=Pre_{C}\left(x_{i}\right)$$

Given BasicVSR’s remarkable achievements in video super-resolution (VSR) facilitated by feature propagation, we employ an identical residual block as a pre-cleaning module. Furthermore, to achieve a more lightweight module, we use a streamlined reconstruction module while maintaining performance. Subsequently, the clean images are forwarded to the VSR network for super-resolution. To guide the training of the image pre-cleaning module, we employ the Charbonnier loss [30] to constrain the output of the pre-cleaning module.

(3) $$y_{i}=S\left(\left\{{\hat{x}}_{i}\right\}\right)$$

### 3.3. Unsupervised Optical Flow Estimator

The feature alignment module in VSR primarily adopts optical flow-based alignment methods. As analyzed in Section 2, the quality of optical flow significantly impacts the performance of these methods. However, previous supervised flow-based methods are limited by the distinction in the domain between synthetic optical flow and real-world optical flow datasets. Consequently, we introduce an unsupervised optical flow estimator [31]. Our purpose is to construct an optical flow network $F_{l}$ capable of accurately estimating motion information $F_{12}^{x}$ from low-quality videos $\left\{x_{1},x_{2}\right\}$.

(4) $$F_{12}^{x}=F_{l}\left(x_{1},x_{2}\right)$$

Based on the fact that the motion information displayed is more accurate between high-quality (HQ) frames $\left\{y_{1},y_{2}\right\}$, we employ the optical flow between HQ video frames to guide the training of the optical flow estimator $F_{l}$. Firstly, we train a teacher optical flow estimator $F_{t}$ on the high-quality frames. Once this optical flow estimator $F_{t}$ converges, it is then frozen and employed as the teacher network in the subsequent phase. $F_{12}^{y}$ represents the optical flow field for two consecutive frames in the HQ videos. As previously mentioned,

(5) $$F_{12}^{y}=F_{t}\left(y_{1},y_{2}\right)$$

and the optical flow information between successive frames of HQ frames is more precise. We employ the more precise $F_{12}^{y}$ as a pseudo-label for the flows $F_{12}^{x}$ and introduce the concept of the distillation loss: the upsampling operation Up ensures that $F_{12}^{x}$ matches the dimensions of $F_{12}^{y}$ in the VSR task.

(6) $$L_{dis}\left(F_{12}^{x},F_{12}^{y}\right)=\sum |F_{12}^{y}-Up\left(F_{12}^{x}\right)|$$

### 3.4. Lightweight Reconstruction Module

In the realm of non-blind VSR, BasicVSR exhibits remarkable performance in the feature alignment module. Inspired by this, to strike a balance between computational complexity and performance in DAVSR, we aim to make its feature alignment module lightweight while maintaining an unchanged performance. Neural networks are composed of blocks, and the feature alignment module within BasicVSR conforms to this architectural convention. Each block is composed of common components, i.e., 3 × 3 convolution, ReLU, and shortcut. To maintain a simple structure, our guiding principle is not to introduce entities unless necessary. ReLU, the activation function employed in the BasicVSR block, is widely adopted in the domain of computer vision. However, recent state-of-the-art (SOTA) methods mostly adopt gated linear units (GLUs) as the activation function. Given their advantages in the low vision field, we contemplated replacing ReLU with a GLU. The formulation for gated linear units is expressed as follows, where X represents the input feature map, σ is a non-linear activation function, *f* and *g* are linear transformers, and ⊙ indicates element-wise multiplication:

(7) Gated(X,σ,f,g)=f(X)⊙σ(g(X))

Although incorporating GLUs into the baseline may enhance the performance, it concurrently raises the block complexity, contradicting our original purpose. To solve this, we optimize the function structure, specifically adopting the GELU:

(8) GELU(x)=xϕ(x)

From Equations (7) and (8), it is evident that the GELU is a specific instance of a GLU. *f*, *g* can be regarded as the same function as input and output and σ is regarded as ϕ. Drawing a parallel, we hypothesize from a different standpoint that GLUs could be seen as a generalization of activation functions, potentially serving as a replacement for nonlinear activation functions. Even if σ is removed, Gated(X)=f(X)⊙g(X) inherently contains nonlinearity. Therefore, we believe that directly partitioning the feature maps along the channel dimension and multiplying them is equivalent to a GLU. This formula is expressed in Equation (9), offering a simplicity that exceeds the implementation of the GELU.

(9) SimpleGate(X,Y)=X⊙Y

**Figure 4:**
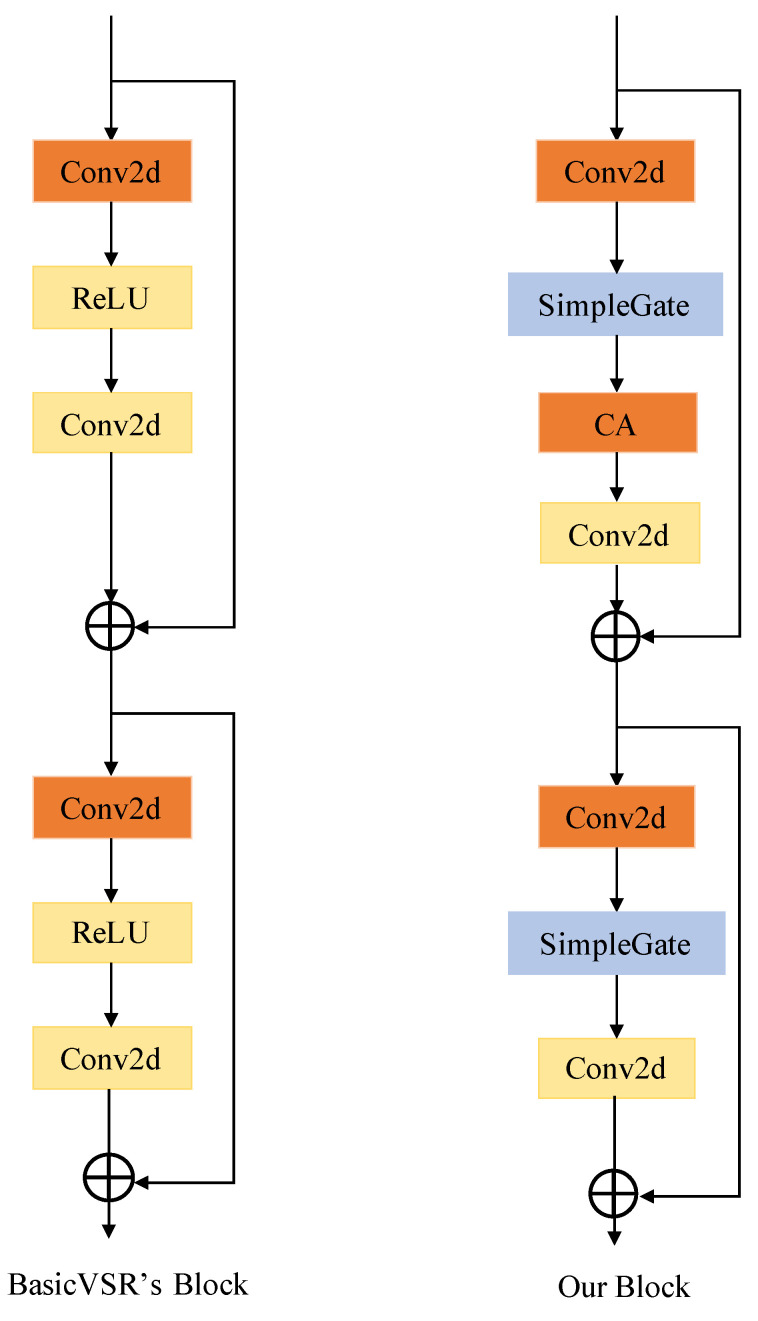
BasicVSR’s block contains the most common elements. Our reconstruction module replaces ReLU with SimpleGate, eliminating the presence of a non-linear activation function, and introduces a channel attention mechanism.

Considering the recent popularity of transformer architectures within the realm of computer vision, the attention mechanism has been widely employed in recent SOTA methods. We incorporate channel attention into the reconstruction block as it enables capturing global information and exhibits efficient computation. A simplified channel attention mechanism can be expressed as follows, where *X* is an input feature map, *pool* represents the global average pooling operation, and *mlp* is a fully connected layer. The global pooling layer conducts dimension compression operations, while *mlp* is used for calculating the channel attention. In comparison to the block structure employed in BasicVSR, our proposed block structure is shown in Figure 4. The forthcoming ablation experiments will confirm that, even with a more lightweight design, our model achieves a comparable performance.

(10) CA(X)=σ(mlp(pool(X)))·X

## 4. Experimental Results

In this section, we compare the proposed method with the state of the art both quantitatively and qualitatively, including image models ESRGAN [32], TDAN [7], IKC [33], and Real-ESGRAN [16] and video models RealVSR [34] and DBVSR [15]. The experimental details are outlined below.

### 4.1. Testing Datasets

**Figure 5:**
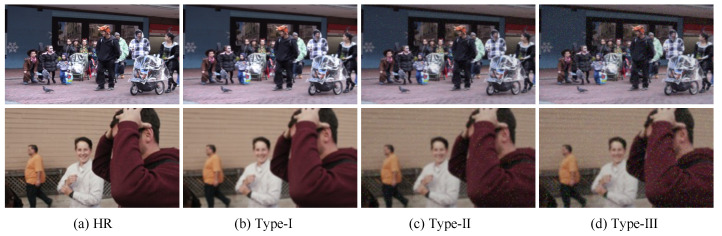
Images with different levels of degradation in vid4 and vimeo-90k.

Existing non-blind VSR methods are assessed on specific synthetic datasets, which cannot reflect the performance of the real-world video resolution enhancement. The testing datasets evaluated by some blind super-resolution methods are only specifically designed synthetic data. For instance, IKC is assessed by synthetic LR images that are blurred and downsampled. As far as we know, a comprehensive low-resolution video dataset, encompassing noise degradations and diverse blurs, is currently unavailable. For quantitative evaluation, we synthesized LR–HR pairs by subjecting the validation datasets to three levels of degradation in vid4 [35] and vimeo-90k [36]. An illustration depicting video frames with different types of degradations is shown in Figure 5. Specifically, the three degradation types for vid4 and vimeo-90k include (1) Type I: a first-order degradation model equipped with blur, noise with Gaussian σ [1, 10], and video compression. It corresponds to slightly corrupted frames. (2) Type II: a first-order degradation model equipped with blur, noise with Gaussian σ [1, 20], resize, and video compression. It corresponds to moderately corrupted frames. (3) Type III: a two-order degradation model, a repeated first-order degradation model, which corresponds to real-world badly corrupted frames. Although the proposed three-level degradation model is not flawless, it strives to cover the entire degradation space of the real world. We contend that the performance of testing datasets can reflect the advantages of our method.

### 4.2. Training Details and Quantitative Metric

All experiments were performed within the PyTorch framework, with training facilitated by NVIDIA 3090 GPUs. Following previous works [15], we employed the REDS dataset [37] for training. For efficiency, we employed the lightweight SR network as our backbone and adaptive degradation processes for simplicity and effectiveness. We adopted the Adam [38] optimizer to train the network. The training process is divided into two stages: Initially, we trained the network using the distillation loss of optical flow and output loss for 300 K iterations with a learning rate of $10^{-4}$. After training the network, we fine-tuned the network with the perceptual loss $L_{perceptual}$ and the adversarial loss $L_{adversarial}$ on different levels of degradation for 200K iterations. The generator was assigned a learning rate of $5\times 10^{-5}$, while the discriminator was set a learning rate of $10^{-4}$. Following common practice, we utilized the widely adopted the PSNR and SSIM as the evaluation metrics. The PSNR (peak signal-to-noise ratio, where higher values indicate a better quality) is commonly used to measure the quality of reconstructed images after compression, typically represented in dB [39]. The SSIM (structural similarity index, where higher values indicate a better quality) is commonly employed to measure the similarity between two images, with its values ranging between 0 and 1 [40].

### 4.3. Comparison to the State of the Art

#### 4.3.1. Quantitative Comparison

Due to the fact that VSR methods demonstrate superior performance exclusively on degraded video frames through bicubic interpolation, and despite the increased complexity in the degradation space of recent blind VSR methods, their performance remains constrained when applied to video frames with varying degrees of degradation in real-world scenarios. To demonstrate the superiority of our degradation-adaptive model, we conduct experimental comparisons on datasets with different levels of degradations. Type III degradation corresponds to videos with severe degradation, type II degradation corresponds to videos with moderate degradation, and type I degradation corresponds to videos with mild degradation. Moreover, these three types of degraded video frames can effectively represent a diverse range of damaged video frames encountered in the real world. Specifically, Table 1 illustrates the restoration performance of various methods under different degradation types, assessed through the evaluative metrics of the PSNR and SSIM. As shown in Table 1, current methods demonstrated superior performance solely under specific degradation conditions, while exhibiting shortcomings in alternative scenarios. In comparison to other methods, our method exhibits q stable and superior performance under three types of degraded images. Meanwhile, as shown in Table 2, a comparison of the inference speed for different networks was conducted. It can be observed that the size of our model (DAVSR) is 36.8% of DBVSR. However, our network’s inference speed is slightly faster compared to this. Moreover, the inference speed is five times faster than RealVSR. Therefore, our network processing speed has an advantage compared to the other two blind video super-resolution algorithms.

Specifically, ESRGAN and RealVSR exhibit a favorable performance on bicubic-downsampled datasets, while experiencing a notable decline in performance on other degraded video frames. For instance, ESRGAN exhibits a performance enhancement of 12% when applied to bicubic-degraded data in comparison to its performance on type I and type II datasets. Even as a blind VSR method, RealVSR exhibits a 9% performance decline on datasets of type III. Recent blind super-resolution methods have shown remarkable restoration effects on severely damaged images. However, a decline in performance is observed when confronted with ’type I’ and ’type II’ degradation, involving mild noise and blurring. Our proposed method, DAVSR, maintains a consistent performance on datasets with different degradation types, exhibiting only a performance decline for type III datasets compared to Real-ESRGAN.

#### 4.3.2. Qualitative Comparison

**Figure 6:**
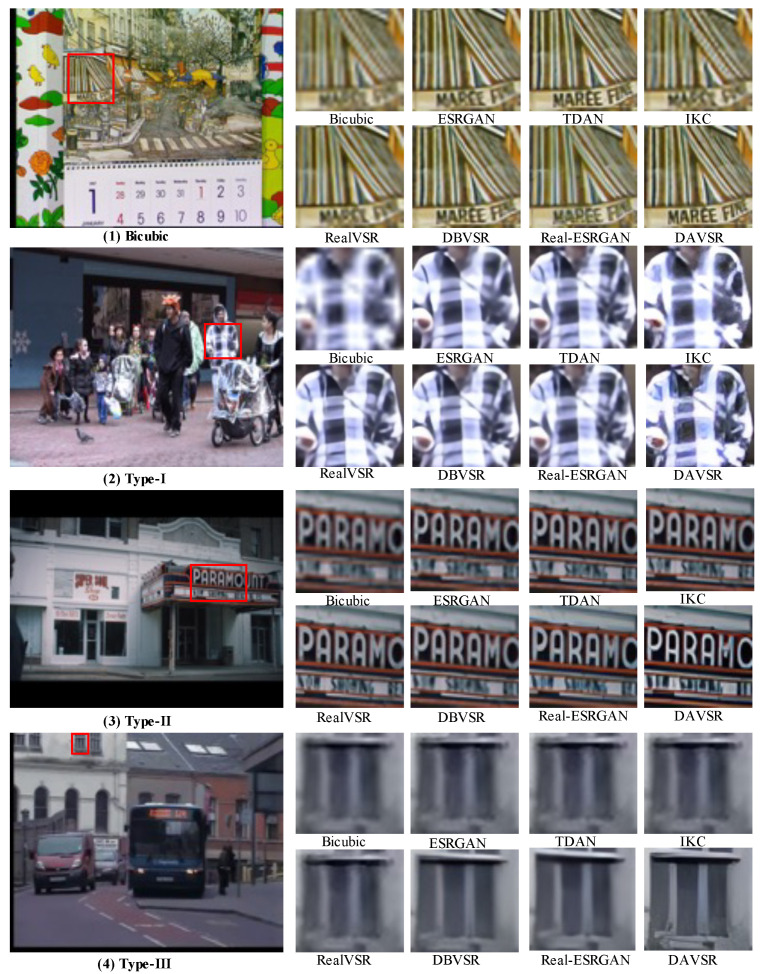
A qualitative comparison of different methods on video frames with diverse degradations. The proposed DAVSR demonstrates superior performance in recovering intricate image structures and achieving enhanced visual quality.

Figure 6 provides a comparative analysis of the qualitative aspects of different methods applied to video frames exhibiting various degradations. Specifically, both RealVSR and Real-ESRGAN fail to generate a satisfactory texture and realistic details on video frames degraded by bicubic downsampling. From the perspective of the restoration results, there is no difference between blind video super-resolution and non-blind video super-resolution networks. This is attributed to the fact that the training data for non-blind super-resolution underwent degradation through bicubic downsampling, resulting in an excellent performance on low-quality video sequences subjected to the same degradation. Conversely, the Real-ESRGAN method was trained on severely degraded datasets. Consequently, when applied to restore mildly damaged video frames, the network tends to amplify noise, resulting in a simultaneous reduction in the accuracy of detail generation. In contrast, our DAVSR is trained on datasets with different degradation types. Therefore, it exhibits a stable performance in restoring video frames with different degrees of degradation. As illustrated in Figure 6(1), our DAVSR achieves restoration closer to the high-resolution (HR) frames and exhibits richer details. (For instance, the outline of the eaves appears more distinct and natural, while the font of the store is sharper and more refined.) Similarly, our network demonstrates a superior restoration performance in video frames with moderate and severe degradation, e.g., in Figure 6(4), the sharp edge of the building window lines.

### 4.4. Effectiveness of Pre-Cleaning Strategy

**Figure 7:**
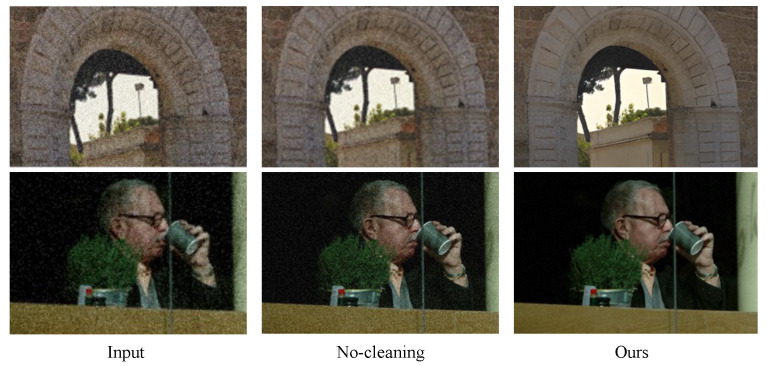
Exploring the effectiveness of the pre-cleaning strategy. The cleaning module plays a crucial role in suppressing artifacts and noise, which is essential for subsequent information propagation in degraded low-resolution images.

We conducted experiments to validate the effectiveness of our pre-processing strategy. Initially, the network was trained with the inclusion of the pre-cleaning model. The degraded video frame sequences underwent optical flow alignment and reconstruction directly without passing through the pre-cleaning module, resulting in temporal redundancy being introduced by the degradation process. Examples are depicted in Figure 7. The network without a cleaning strategy lacks sufficient suppression of artifacts and noise, resulting in perceptually impactful noise in the recovered images. This is attributed to the network without a cleaning strategy, which fails to eliminate redundant temporal information, consequently amplifying noise and artifacts. Consequently, it yields a comparatively diminished efficacy in restoration compared to the cleaning network.

**Figure 8:**
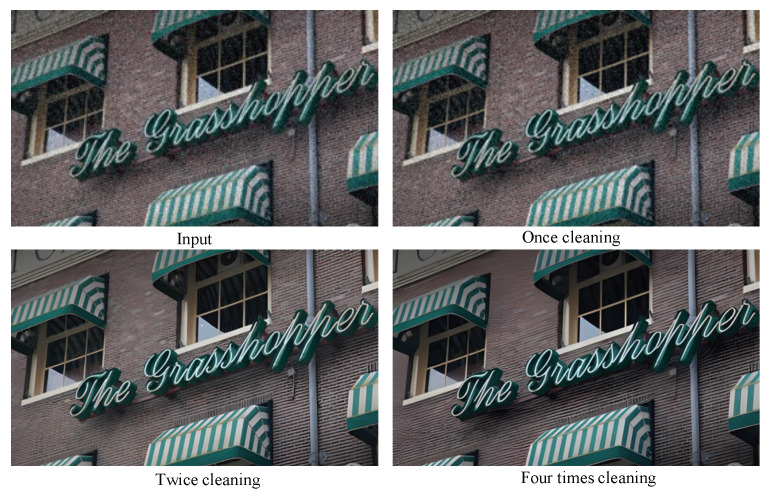
Exploring the impact of the number of pre-cleaning module iterations. For mildly degraded images, our network achieves optimal results when applying the cleaning strategy twice.

Furthermore, we kept the cleaning module and explored the influence of the number of cleaning iterations on the removal of temporal information. The restoration results for images with degradation type I are shown in Figure 8. On the one hand, when the cleaning module is applied only once, the noise is not removed and blurriness persists. On the other hand, employing the cleaning module four times results in excessive noise removal, leading to an unrealistic over-smoothing of images. We observe that for mildly degraded images, applying the cleaning module twice is optimal. As anticipated, for images representing degradation type III, characterized by severe degradation, the application of the pre-cleaning module twice is insufficient. Our observations indicate that, in most scenarios, a maximum of three iterations suffices. We ascertain that for images exhibiting degradation types I and II, a two-fold application of the pre-processing module will suffice. Conversely, for images representing degradation type III, a three-fold application of the module is deemed adequate.

### 4.5. Ablation Studies

#### 4.5.1. Unsupervised Optical Flow Estimator

To validate the effectiveness of our unsupervised optical flow estimator, we retrained a supervised optical flow estimator, specifically SpyNet. This is a widely used optical flow network in the state of the art (SOTA), such as BasicVSR. As shown in Table 3, SpyNet trained with an unsupervised approach can outperform the supervised counterpart by 0.13 dB on the vid4 dataset. Furthermore, on the vimeo-90k dataset, the performance surpasses 0.15 dB. The experiments conducted on various datasets suggest that the unsupervised optical flow alignment method achieves better frame alignment results. Additionally, this improvement is attributed to the utilization of optical flow between high-quality video frames to guide the training of our optical flow estimator.

#### 4.5.2. Lightweight Reconstruction Module

From BasivVSR’s Block to our Block: Our reconstruction module is composed of concatenating multiple blocks. A comparison of the reconstruction block between BasicVSR and our DAVSR is illustrated in Figure 4. Notably, we have introduced a channel attention mechanism and replaced the ReLU activation with a simplified SimpleGate in our block structure. Ablation studies were conducted on the vid4 and vimeo-90k datasets. We employed the training approach mentioned in Section 4.2 for training. The effectiveness of channel attention and SimpleGate is demonstrated in Table 4. A performance improvement is observed when either replacing ReLU in the reconstruction module with SimpleGate (SG) or adding channel attention (CA) to the reconstruction module for both vid4 and vimeo-90k datasets.

Number of Blocks: Due to the fact that our reconstruction module is formed by concatenating multiple blocks, we investigated the effect of the number of blocks in the reconstruction module on its restoration effectiveness in Table 5. To ensure the lower bounds of the restoration performance within the reconstruction module, we designed an experimental setup with the number of blocks ranging from 10 to 30. It is evident from our experiments that augmenting the number of blocks to 30 does not yield a substantial enhancement in network performance. On the contrary, the model params increase by 50%. Clearly, the benefits of increasing the number of blocks are limited. Therefore, we set the number of blocks in the DAVSR network to 20, reaching the optimal performance for the reconstruction module.

## 5. Conclusions

In this paper, we propose degradation-adaptive blind video super-resolution, namely DVASR, to handle various types of degraded video frames in the real world. In order to capture various degradation processes in the real world, we propose a degradation-adaptive model for modeling. The proposed DAVSR exhibits favorable restoration when applied to video frames with different levels of degradation. Moreover, we utilize a pre-cleaning strategy to mitigate the adverse effects stemming from temporal redundancy between video frames. Meanwhile, we employ a lightweight reconstruction module to balance the computational complexity and the performance of DAVSR. Extensive experiments on several benchmark datasets achieve a state-of-the-art SR performance.

Due to the significantly higher computational complexity of super-resolution networks compared to image classification and object detection networks, in the future, we will explore more lightweight architectures and real-time super-resolution networks.

## Figures and Tables

**Table 1:** Comparison of different methods’ quantitative performance on vid4 datasets with different degradations. The optimal results are indicated in bold. ‘Types I’, ‘II’, and ‘III’ are employed to signify datasets characterized by mild, medium, and severe degradations, respectively. ‘Bicubic’ denotes the vid4 dataset with bicubic interpolation.

D-Type	ESRGAN	TDAN	IKC	RealVSR	DBVSR	Real-ESRGAN	DAVSR
Bicubic	25.03/0.721	26.16/0.782	24.89/0.712	26.65/0.795	26.87/0.805	26.93/0.809	**27.04/0.816**
Type-I	20.36/0.522	26.20/0.783	24.72/0.703	26.51/0.784	26.74/0.798	26.84/0.803	**26.95/0.810**
Type-II	21.98/0.587	25.87/0.770	24.55/0.696	26.44/0.779	26.66/0.796	26.78/0.801	**26.83/0.803**
Type-III	23.21/0.604	23.14/0.602	23.08/0.597	24.12/0.652	24.27/0.658	**25.45/0.751**	25.32/0.744

**Table 2:** Comparison of different methods’ inference speeds with an input size of 180×320.

Method	TDAN	RealVSR	DBVSR	Real-ESRGAN	DAVSR
Params (M)	1.97	2.7	25.5	16.7	9.4
Runtime (ms)	138	1082	239	149	216

**Table 3:** A comparative analysis of unsupervised and supervised training approaches for SpyNet networks in video super-resolution. “Sup” represents supervised training; “Unsup” represents unsupervised training.

Dataset	Vid4		Vimeo-90k	
Method	Sup	Unsup	Sup	Unsup
PSNR(db)	26.91	27.04	34.78	34.93
SSIM	0.804	0.816	0.929	0.937

**Table 4:** The effectiveness of channel attention (CA) and SimpleGate (SG) has been validated. In the table, Original Block refers to the reconstruction module of BasicVSR.

	ReLU → SG	CA	Vid4 PSNR SSIM	Vimeo-90k PSNR SSIM
Original Block			26.82/0.803	33.74/0.925
↓	✓		26.98/0.812	34.56/0.931
		✓	26.92/0.808	34.37/0.929
Our Block	✓	✓	27.04/0.816	34.93/0.937

**Table 5:** The impact of residual block quantity on the performance of the reconstruction module.

	Number of Blocks	Params (M)	Vid4 PSNR SSIM	Vimeo-90k PSNR SSIM
DAVSR	10	2.82	26.73/0.798	33.25/0.916
	20	5.64	27.04/0.816	34.93/0.937
	30	8.46	27.05/0.816	35.05/0.938

## Data Availability

The data presented in this study are available from the authors upon reasonable request.
